# Social Support and the Association Between Certain Forms of Violence and Harassment and Suicidal Ideation Among Transgender Women — National HIV Behavioral Surveillance Among Transgender Women, Seven Urban Areas, United States, 2019*–*2020

**DOI:** 10.15585/mmwr.su7301a7

**Published:** 2024-01-25

**Authors:** Patrick C. Eustaquio, Evelyn Olansky, Kathryn Lee, Ruthanne Marcus, Susan Cha, National HIV Behavioral Surveillance Among Transgender Women Study Group, National HIV Behavioral Surveillance Among Transgender Women Study Group, Narquis Barak, Kathleen A. Brady, Sarah Braunstein, Jasmine Davis, Sara Glick, Andrea Harrington, Jasmine Lopez, Yingbo Ma, Aleks Martin, Genetha Mustaafaa, Tanner Nassau, Gia Olaes, Jennifer Reuer, Alexis Rivera, William T. Robinson, Ekow Kwa Sey, Sofia Sicro, Brittany Taylor, Dillon Trujillo, Erin Wilson, Pascale Wortley

**Affiliations:** ^1^Division of HIV Prevention, National Center for HIV, Viral Hepatitis, STD, and TB Prevention, CDC, Atlanta, Georgia; ^2^Oak Ridge Institute for Science and Education, Oak Ridge, Tennessee; CrescentCare; Philadelphia Department of Public Health; New York City Department of Health and Mental Hygiene; CrescentCare; University of Washington, School of Medicine, Division of Allergy and Infectious Diseases, Public Health – Seattle & King County, HIV/STD Program; Philadelphia Department of Public Health; New York City Department of Health and Mental Hygiene; Los Angeles County Department of Public Health; Public Health – Seattle & King County, HIV/STD Program; Georgia Department of Public Health; Philadelphia Department of Public Health; Los Angeles County Department of Public Health; Washington State Department of Health; New York City Department of Health and Mental Hygiene; Louisiana State University Health Science Center in New Orleans – School of Public Health, Louisiana Office of Public Health STD/HIV/Hepatitis Program; Los Angeles County Department of Public Health; San Francisco Department of Public Health; Georgia Department of Public Health; San Francisco Department of Public Health; San Francisco Department of Public Health; Georgia Department of Public Health.

## Abstract

Violence and harassment toward transgender women are associated with suicidal thoughts and behaviors, and social support might moderate such association. This analysis explored the association between certain forms of violence and harassment and suicidal ideation and moderation by social support. Better understanding of these associations could guide mental health services and structural interventions appropriate to lived experiences of transgender women. This cross-sectional analysis used data from CDC’s National HIV Behavioral Surveillance Among Transgender Women. During 2019–2020, transgender women were recruited via respondent-driven sampling from seven urban areas in the United States for an HIV biobehavioral survey. The association between experiencing certain forms of violence and harassment (i.e., gender-based verbal and physical abuse or harassment, physical intimate partner abuse or harassment, and sexual violence) and suicidal ideation was measured using adjusted prevalence ratios and 95% CIs generated from log-linked Poisson regression models controlling for respondent-driven sampling design and confounders. To examine moderation, the extents of social support from family, friends, and significant others were assessed for interaction with certain forms of violence and harassment; if p interaction was <0.05, stratified adjusted prevalence ratios were presented. Among 1,608 transgender women, 59.7% experienced certain forms of violence and harassment and 17.7% reported suicidal ideation during the past 12 months; 75.2% reported high social support from significant others, 69.4% from friends, and 46.8% from family. Experiencing certain forms of violence and harassment and having low-moderate social support from any source was associated with higher prevalence of suicidal ideation. Social support from family moderated the association between experiencing certain forms of violence and harassment and suicidal ideation (p interaction = 0.01); however, even in the presence of high family social support, experiencing certain forms of violence and harassment was associated with higher prevalence of suicidal ideation. Social support did not completely moderate the positive association between experiencing violence and harassment and suicidal ideation. Further understanding of the social support dynamics of transgender women might improve the quality and use of social support. Policymakers and health care workers should work closely with transgender women communities to reduce the prevalence of violence, harassment, and suicide by implementing integrated, holistic, and transinclusive approaches.

## Introduction

A high proportion of transgender persons considered or attempted suicide at some point during their lives, often higher than in the general population (*1*), with notably higher prevalence among young transgender persons and transgender persons from racial and ethnic minority groups (*2*–*5*). In the 2015 U.S. Transgender Survey, 82% of respondents ever considered and 40% ever attempted suicide; 48% of respondents considered and 7% attempted suicide during the past year (*2*). Further, transgender women are more likely to report suicidal thoughts than transgender men, nonbinary persons, and other gender diverse groups (*6*). On the basis of CDC’s National HIV Behavioral Surveillance Among Transgender Women (NHBS-Trans) during 2019–2020, a total of 18% considered and 4% attempted suicide during the past 12 months (*7*). 

Similarly prevalent among transgender persons are experiences of violence and harassment. Studies reported a wide range in lifetime violence among transgender persons (7%–89%), which limits understanding of the true prevalence in this population (*8*). Violence and harassment against transgender persons come in many forms (e.g., verbal, physical, sexual, occupational, economic, and emotional) and from many sources (e.g., interpersonal, partner or nonpartner, and structural) (*8*). Particularly, transgender women are more frequently victimized than other transgender and gender diverse groups (*9*); such is often attributed to transmisogyny, an intersection stigma based on trans identity and feminine expression (*10*). Violence and harassment have been associated with higher risk for HIV infection (*11*), mental health conditions (*5**,**12*), and death, often from suicide (*13**–**15*). The association between violence and harassment and increased suicidal thoughts and behaviors among transgender persons is consistent across studies (*3**,**13**,**14**,**16**,**17*). Social support might attenuate the association, although studies exploring such a hypothesis among transgender persons are scant and violence and harassment were not analyzed separately from other adverse social experiences (*15**,**18**–**20*).

Scientific gaps remain because most previous studies were not focused on transgender women and have examined violence and harassment, suicidal ideation, and social support separately. This analyses in this report examined the association between experiences of certain forms of violence and harassment and suicidal ideation among transgender women and explored the moderation of the association by perceived social support. A thorough understanding of the intersectionality of these factors could help guide recommendations for mental health services and structural interventions tailored to lived experiences of transgender women.

## Methods

### Data Source

This report includes survey data from NHBS-Trans conducted by CDC during June 2019–February 2020 to assess behavioral risks, prevention usage, and HIV prevalence. Eligible participants completed an interviewer-administered questionnaire and were offered HIV testing. Information and referrals to appropriate services, which were identified as available and acceptable to the population during formative assessment, were provided to participants who reported experiences of violence and harassment and suicidal thoughts and behaviors. Additional information about NHBS-Trans eligibility criteria, data collection, and biologic testing is available in the overview and methodology report of this supplement (*21*). The NHBS-Trans protocol questionnaire and documentation are available at https://www.cdc.gov/hiv/statistics/systems/nhbs/methods-questionnaires.html#trans.

Applicable local institutional review boards in each participating project area approved NHBS-Trans activities. The final NHBS-Trans sample included 1,608 transgender women in seven urban areas in the United States (Atlanta, Georgia; Los Angeles, California; New Orleans, Louisiana; New York City, New York; Philadelphia, Pennsylvania; San Francisco, California; and Seattle, Washington) recruited using respondent-driven sampling. This activity was reviewed by CDC, deemed not research, and was conducted consistent with applicable Federal law and CDC policy.^*^

### Measures

The gender minority stress model (*14*) underpinned the conceptual framework for the analysis (Figure). This model posits that being part of a gender minority contributes to multiple stressors, including violence and harassment, that negatively affect health outcomes, including suicidal ideation, among transgender women (*14*). Social support was analyzed as a resilience factor that could moderate the association between violence and suicidal ideation.

**FIGURE:**
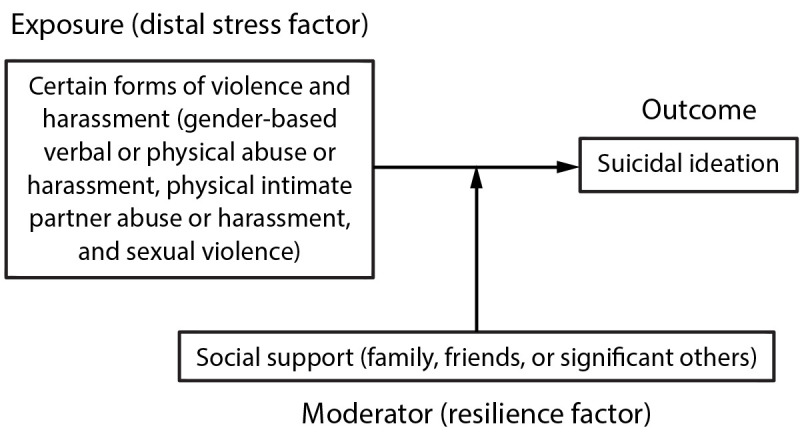
Conceptual framework of analysis based on the gender minority stress model — National HIV Behavioral Surveillance Among Transgender Women, seven urban areas,* United States, 2019–2020 **Source:** Testa RJ, Michaels MS, Bliss W, Rogers ML, Balsam KF, Joiner T. Suicidal ideation in transgender people: gender minority stress and interpersonal theory factors. J Abnorm Psychol 2017;126:125–36. * Atlanta, GA; Los Angeles, CA; New Orleans, LA; New York City, NY; Philadelphia, PA; San Francisco, CA; and Seattle, WA.

The outcome assessed was suicidal ideation during the past 12 months (Table 1). The exposure assessed was experiences with certain forms of violence and harassment, which was operationally defined as gender-based verbal or physical abuse or harassment, physical abuse or harassment by an intimate partner, or sexual violence during the past 12 months. The creation of this composite variable (*22*) was determined by the high co-occurrence of multiple forms of violence and harassment among transgender populations among different studies (*8*) and in the current analytical sample. The moderator assessed was perceived social support, measured using the Multidimensional Scale of Perceived Social Support, dichotomized as low-moderate (mean <3.57) and high (mean ≥3.57) (Cronbach’s alpha = 0.97) (*23*). All three social support subscales (family, friends, and significant others) were assessed separately. The instrument demonstrated good construct validity and internal consistency among transgender persons (*15*). Confounding factors, determined a priori (*15**,**16**,**24*), included age, race and ethnicity, poverty, education, HIV testing result, hormonal and surgical gender-affirmation status, illicit drug use, disability, incarceration, and homelessness.

**TABLE 1:** Variables, questions, and analytic coding for social support and the association between certain forms of violence and harassment and suicidal ideation among transgender women — National HIV Behavioral Surveillance Among Transgender Women, seven urban areas,* United States, 2019–2020

Variable	Question	Analytic coding
Suicidal ideation	At any time in the past 12 months, did you seriously think about trying to kill yourself?	Yes or no
Certain forms of violence and harassment^†^	Any reports of gender-based verbal and physical abuse or harassment, physical intimate partner abuse or harassment, and sexual violence in the past 12 months.	Yes or no
Verbal abuse or harassment	In the past 12 months, have you been verbally abused or harassed because of your gender identity or presentation?	Yes or no
Physical abuse or harassment	In the past 12 months, have you been physically abused or harassed because of your gender identity or presentation?	Yes or no
Physical intimate partner abuse or harassment	In the past 12 months, have you been physically abused or harassed by a sexual partner?	Yes or no
Sexual violence	In the past 12 months, have you been forced to have sex when you did not want to? By forced, I mean physically forced or verbally threatened. By sex, I mean any sexual contact.	Yes or no
Social support from family	5-point Likert scale (from strongly agree to strongly disagree) • My family really tries to help me. Do you … • I get the emotional help and support I need from my family. Do you … • I can talk about my problems with my family. Do you … • My family is willing to help me make decisions. Do you …	Low-moderate (mean <3.57) or high (mean ≥3.57)
Social support from friends	5-point Likert scale (from strongly agree to strongly disagree) • My friends really try to help me. Do you … • I can count on my friends when things go wrong. Do you … • I have friends with whom I can share my joys and sorrows. Do you … • I can talk about my problems with my friends. Do you …	Low-moderate (mean <3.57) or high (mean ≥3.57)
Social support from significant others	5-point Likert scale (from strongly agree to strongly disagree) • There is a special person who is around when I am in need. Do you … • There is a special person with whom I can share joys and sorrows. Do you … • I have a special person who is a real source of comfort to me. Do you … • There is a special person in my life who cares about my feelings. Do you …	Low-moderate (mean <3.57) or high (mean ≥3.57)
Age	What is your date of birth?	18–24 yrs, 25–29 yrs, 30–39 yrs, 40–49 yrs, or >50 yrs
Race and ethnicity^§^	Do you consider yourself to be of Hispanic, Latino/a, or Spanish origin? Which racial group or groups do you consider yourself to be in? You may choose more than one option.	Black or African American, White, Hispanic, or other
Poverty^¶^	What was your household income last year from all sources before taxes? Including yourself, how many people depended on this income?	Above Federal poverty level or at or below Federal poverty level
Education	What is the highest level of education you completed?	<High school, high school diploma or equivalent, or >high school
GAHT status	Have you ever taken hormones for gender transition or affirmation? Are you currently taking hormones for gender transition or affirmation? Would you like to take hormones for gender transition or affirmation?	Do not want to take GAHT, currently taking GAHT, or want to take GAHT
Gender-affirming surgery status	Have you ever had any type of surgery for gender transition or affirmation? Do you plan or want to get additional surgeries for gender transition or affirmation? Do you want to have surgery for gender transition or affirmation?	No unmet gender-affirming surgery need, had procedures, or wants gender-affirming surgery but has not received procedures
Disability**	Are you deaf or do you have serious difficulty hearing? Are you blind or do you have serious difficulty seeing, even when wearing glasses? Because of a physical, mental, or emotional condition, do you have serious difficulty concentrating, remembering, or making decisions? Do you have serious difficulty walking or climbing stairs? Do you have difficulty dressing or bathing? Because of a physical, mental, or emotional condition, do you have difficulty doing errands alone, such as visiting a doctor's office or shopping?	Yes or no
Illicit drug use (excluding marijuana)	Have you ever in your life shot up or injected any drugs other than those prescribed for you? How many days or months or years ago did you last inject? In the past 12 months, have you used any drugs that were not prescribed for you and that you did not inject?	Yes or no
Incarceration	Have you ever been held in a detention center, jail, or prison for more than 24 hours? During the past 12 months, have you been held in a detention center, jail, or prison for more than 24 hours?	Never incarcerated, incarcerated in the past 12 months, or incarcerated >12 months ago
Homelessness	In the past 12 months, have you been homeless at any time? By homeless, I mean you were living on the street, in a shelter, in a single room occupancy hotel (SRO), or in a car. Are you currently homeless?	Currently homeless, was homeless in the past 12 months but not currently, or no homelessness in the past 12 months

**Abbreviation:** GAHT = gender-affirming hormonal therapy.

* Atlanta, GA; Los Angeles, CA; New Orleans, LA; New York City, NY; Philadelphia, PA; San Francisco, CA; and Seattle, WA.

^†^ Not a questionnaire item in National HIV Behavioral Surveillance Among Transgender Women. This is a composite analysis variable from the responses in actual survey questions on verbal abuse or harassment, physical abuse or harassment, physical intimate partner abuse or harassment, and sexual violence.

^§^ Persons of Hispanic or Latina (Hispanic) origin might be of any race but are categorized as Hispanic; all racial groups are non-Hispanic.

^¶^ 2019 Federal poverty level thresholds were calculated on the basis of U.S. Department of Health and Human Services Federal poverty level guidelines (https://aspe.hhs.gov/topics/poverty-economic-mobility/poverty-guidelines/prior-hhs-poverty-guidelines-federal-register-references/2019-poverty-guidelines).

** To assess difficulty in six basic domains of functioning (hearing, vision, cognition, walking, self-care, and independent living), based on U.S. Department of Health and Human Services disability data standard (https://aspe.hhs.gov/reports/hhs-implementation-guidance-data-collection-standards-race-ethnicity-sex-primary-language-disability-0).

### Analysis

The association between certain forms of violence and harassment and suicidal ideation was examined using log-linked Poisson regression models with generalized estimating equations with an exchangeable correlation matrix, with robust variance estimators. Bivariable models were used to determine factors associated with suicidal ideation, and the associations were described as crude prevalence ratios with 95% CIs. Multivariable models, controlled for confounding factors, were used to examine the association of certain forms of violence and harassment and social support with suicidal ideation, and the associations were described as adjusted prevalence ratios with 95% CIs. All bivariable and multivariable models accounted for the respondent-driven sampling methodology by adjusting for network size and city and by clustering on recruitment chains. Moderation by social support subscales were assessed using interaction terms of dichotomized social support subscale scores and certain forms of violence and harassment in multiplicative scale in separate multivariable models (*25*). The interaction between family social support and certain forms of violence and harassment was statistically significant (p<0.05); hence, stratified adjusted prevalence ratios by extent of family social support were calculated (*25*). Statistical analyses were conducted using SAS (version 9.4; SAS Institute).

## Results

Among transgender women in the sample (N = 1,608), many were aged <40 years (59.5%), were Hispanic or Latina (Hispanic) (40.0%) or Black or African American (Black) (35.4%), lived at or below the Federal poverty level (62.7%), were ever incarcerated (58.1%; 17.2% during the past 12 months), and had experienced homelessness during the past 12 months (41.6%) (Table 2). (Persons of Hispanic origin might be of any race but are categorized as Hispanic; all racial groups are non-Hispanic.) Most were currently taking gender-affirming hormonal therapy (71.5%) and wanted gender-affirming surgery but had not received procedures (52.2%); 41.0% tested positive for HIV. During the past 12 months, 59.7% experienced certain forms of violence and harassment: 53.4% reported gender-based verbal abuse or harassment, 26.6% reported gender-based physical abuse or harassment, 15.3% reported being physically abused or harassed by an intimate partner, and 14.8% reported sexual violence (not mutually exclusive). Among all participants, 75.2% reported high social support from significant others, 69.4% from friends, and 46.8% from family.

**TABLE 2:** Number and percentage of transgender women experiencing certain forms violence and harassment, by reported suicidal ideation and selected characteristics **—** National HIV Behavioral Surveillance Among Transgender Women, seven urban areas,* United States, 2019–2020

Characteristic	Total (N = 1,608)	Reported suicidal ideation during the past year			
		Yes	No	Crude PR (95% CI)^§^	Adjusted PR (95% CI)^§^
	No.^†^ (%)	No. (%)	No. (%)		
**Overall**	**1,608 (100)**	**284 (17.7)**	**1,318 (82.3)**	**NA**	**NA**
**Age group, yrs**					
18–24	**190 (11.8)**	50 (26.3)	138 (72.6)	1.00 (Ref)	NA
25***–***29	**306 (19.0)**	69 (22.5)	235 (76.8)	0.85 (0.62–1.17)	NA
30***–***39	**462 (28.7)**	85 (18.4)	376 (81.4)	0.71 (0.55–0.93)^¶^	NA
40***–***49	**307 (19.1)**	44 (14.3)	263 (85.7)	0.56 (0.38–0.81)^¶^	NA
≥50	**343 (21.3)**	36 (10.5)	306 (89.2)	0.41 (0.27–0.6)^¶^	NA
Race and ethnicity**					
Black or African American	**569 (35.4)**	74 (13.0)	492 (86.5)	0.31 (0.25–0.38)^¶^	NA
White	**180 (11.2)**	74 (41.1)	105 (58.3)	1.00 (Ref)	NA
Other	**213 (13.2)**	30 (14.1)	181 (85.0)	0.36 (0.23–0.56)^¶^	NA
Hispanic or Latina	**643 (40.0)**	106 (16.5)	537 (83.5)	0.39 (0.32–0.48)^¶^	NA
**Poverty^††^**					
Above the Federal poverty level	**585 (36.4)**	104 (17.8)	479 (81.9)	1.00 (Ref)	NA
At or below the Federal poverty level	**1,008 (62.7)**	175 (17.4)	830 (82.3)	0.93 (0.76–1.13)	NA
**Education**					
<High school	**347 (21.6)**	44 (12.7)	303 (87.3)	1.00 (Ref)	NA
High school diploma or equivalent	**596 (37.1)**	110 (18.5)	484 (81.2)	1.46 (1.03–2.07)^¶^	NA
>High school	**521 (32.4)**	106 (20.3)	412 (79.1)	1.63 (1.23–2.17)^¶^	NA
**GAHT status**					
Do not want to take GAHT	**121 (7.5)**	12 (9.9)	109 (90.1)	1.00 (Ref)	NA
Currently taking GAHT	**1,149 (71.5)**	200 (17.4)	944 (82.2)	1.91 (0.87–4.22)	NA
Want to take GAHT	**317 (19.7)**	68 (21.5)	248 (78.2)	2.26 (0.99–5.14)	NA
**Gender-affirming surgery status**					
No unmet need	**448 (27.9)**	59 (13.2)	388 (86.6)	1.00 (Ref)	NA
Had procedures, wants more procedures	**232 (14.4)**	34 (14.7)	197 (84.9)	1.18 (0.85–1.63)	NA
Wants but has not received procedures	**840 (52.2)**	173 (20.6)	663 (78.9)	1.53 (1.12–2.09)^¶^	NA
**Confirmed HIV status^§§^**					
Negative	**902 (56.1)**	198 (22.0)	699 (77.5)	1.00 (Ref)	NA
Positive	**659 (41.0)**	82 (12.4)	576 (87.4)	0.57 (0.44–0.73)^¶^	NA
**Disability^¶¶^**					
No	**747 (46.5)**	79 (10.6)	667 (89.3)	1.00 (Ref)	NA
Yes	**853 (53.0)**	202 (23.7)	646 (75.7)	2.27 (1.76–2.93)^¶^	NA
Illicit drug use***					
No	**947 (58.9)**	135 (14.3)	811 (85.6)	1.00 (Ref)	NA
Yes	**657 (40.9)**	148 (22.5)	504 (76.7)	1.58 (1.29–1.95)^¶^	NA
**Incarceration^†††^**					
Never incarcerated	**670 (41.7)**	132 (19.7)	534 (79.7)	1.00 (Ref)	NA
Incarcerated >12 months ago	**658 (40.9)**	100 (15.2)	557 (84.7)	0.77 (0.63–0.95)^¶^	NA
Incarcerated ≤12 months ago	**277 (17.2)**	50 (18.1)	226 (81.6)	0.86 (0.66–1.12)	NA
**Homelessness**					
No homelessness during past 12 months	**936 (58.2)**	131 (14.0)	804 (85.9)	1.00 (Ref)	NA
Was homeless during past 12 months but not currently	**306 (19.0)**	58 (19.0)	247 (80.7)	1.35 (0.97–1.89)	NA
Currently homeless	**364 (22.6)**	94 (25.8)	266 (73.1)	1.80 (1.47–2.2)^¶^	NA
**Certain forms of violence and harassment^§§§^**					
Did not experience	**646 (40.2)**	61 (9.4)	584 (90.4)	1.00 (Ref)	1.00 (Ref)
Experienced	**960 (59.7)**	221 (23.0)	734 (76.5)	2.34 (1.64–3.35)^¶^	1.61 (1.21–2.15)^¶^
**Family social support**					
High	**752 (46.8)**	82 (10.9)	670 (89.1)	1.00 (Ref)	1.00 (Ref)
Low-moderate	**853 (53.0)**	201 (23.6)	646 (75.7)	2.17 (1.68–2.81)^¶^	1.62 (1.27–2.06)^¶^
**Friend social support**					
High	**1,116 (69.4)**	186 (16.7)	926 (83.0)	1.00 (Ref)	1.00 (Ref)
Low-moderate	**491 (30.5)**	98 (20.0)	391 (79.6)	1.19 (1.01–1.39)^¶^	1.20 (1.01–1.43)^¶^
**Significant other social support**					
High	**1,209 (75.2)**	191 (15.8)	1,015 (84.0)	1.00 (Ref)	1.00 (Ref)
Low-moderate	**399 (24.8)**	93 (23.3)	303 (75.9)	1.44 (1.19–1.75)^¶^	1.20 (1.03–1.42)^¶^

**Abbreviations:** GAHT = gender-affirming hormonal therapy; NA = not applicable; PR = prevalence ratio; Ref = referent group.

* Atlanta, GA; Los Angeles, CA; New Orleans, LA; New York City, NY; Philadelphia, PA; San Francisco, CA; and Seattle, WA.

^†^ Numbers might not sum to 1,608, and column percentages might not sum to 100% because of missing values.

^§^ All bivariable and multivariable models were controlled for city and network size and accounted for clustering by respondent-driven sampling recruitment chain. The multivariable models for the exposure and each moderator variables all had separate models and were adjusted for age, race and ethnicity, education, poverty, GAHT status, gender-affirming surgery status, disability, HIV status, incarceration, illicit drug use (excluding marijuana), and homelessness.

^¶^ Statistically significant at p<0.05.

** Persons of Hispanic or Latina (Hispanic) origin might be of any race but are categorized as Hispanic; all racial groups are non-Hispanic. “Other” includes persons who identified with non-Hispanic ethnicity and identified as American Indian/Alaska Native, Native Hawaiian or other Pacific Islander, Asian, or multiracial (i.e., more than one racial group).

^††^ 2019 Federal poverty level thresholds were calculated on the basis of U.S. Department of Health and Human Services Federal poverty level guidelines (https://aspe.hhs.gov/topics/poverty-economic-mobility/poverty-guidelines/prior-hhs-poverty-guidelines-federal-register-references/2019-poverty-guidelines).

^§§^ Participants with reactive rapid National HIV Behavioral Surveillance HIV test result supported by a second rapid test or supplemental laboratory-based testing.

^¶¶^ Serious difficulty hearing, seeing, doing cognitive tasks, walking or climbing stairs, dressing or bathing, or doing errands alone, based on U.S. Department of Health and Human Services disability data standard (https://aspe.hhs.gov/reports/hhs-implementation-guidance-data-collection-standards-race-ethnicity-sex-primary-language-disability-0).

*** Excludes marijuana.

^†††^ Held in a detention center, jail, or prison for >24 hours.

^§§§^ Any reports of gender-based verbal and physical abuse or harassment, physical intimate partner abuse or harassment, and sexual violence during the past 12 months.

During the past 12 months, 17.7% reported suicidal ideation. The prevalence of suicidal ideation was higher among those who were aged 18–24 years, White, had at least a high school education, had an unmet need for gender-affirming surgery, had HIV-negative test results, reported drug use, have a disability, were currently experiencing homelessness, did not report a history of incarceration, reported low-moderate social support from any source, and experienced certain forms of violence and harassment (p<0.05) (Table 2). In the multivariable analyses, both experiencing certain forms of violence and harassment and having low-moderate social support from any source were associated with higher prevalence of suicidal ideation.

The interaction between social support from family and experiencing certain forms of violence and harassment was significant (p interaction = 0.01) (Table 3). However, even among those with high family social support, certain forms of violence and harassment were significantly associated with increased prevalence of suicidal ideation. The interactions between social support from friends and from significant others and experiencing certain forms of violence and harassment were not statistically significant. 

**TABLE 3:** Association between suicidal ideation and experiences of certain forms of violence and harassment* and the moderating effect of family social support — National HIV Behavioral Surveillance Among Transgender Women, seven urban areas,^†^ United States, 2019–2020

Family social support	Did not experience certain forms of violence and harassment			Experienced certain forms of violence and harassment			Experiences of certain forms of violence and harassment within the strata of social support aPR (95% CI)**^,††^
	No. with SI^§,¶,^**	No. without SI^§,¶,^**	aPR (95% CI)**^,††^	No. with SI^§,¶,^**	No. without SI^§,¶,^**	aPR (95% CI)**^,††^	
High	18	371	1.00 (Ref)	63	299	2.60 (1.63–4.16)	2.60 (1.63–4.16)
Low-moderate	43	211	2.83 (1.73–4.63)	157	435	3.24 (2.00–5.24)	1.15 (0.81–1.61)

**Abbreviations:** aPR = adjusted prevalence ratio; SI = suicidal ideation.

* Any reports of gender-based verbal and physical abuse or harassment, physical intimate partner abuse or harassment, and sexual violence during the past 12 months

^†^ Atlanta, GA; Los Angeles, CA; New Orleans, LA; New York City, NY; Philadelphia, PA; San Francisco, CA; and Seattle, WA.

^§^ N = 1,608.

^¶^ Numbers might not sum to 1,608 because of missing values.

** Log-linked Poisson regression models using generalized estimating equation with an exchangeable correlation matrix and robust variance estimators with a significant interaction term between family social support and certain forms of violence and harassment (p interaction = 0.01)

^††^ Models were adjusted for respondent-driven sampling design and confounding factors, including age, race and ethnicity, education, poverty, gender affirming hormonal therapy status, gender affirming surgery status, disability, HIV status, incarceration, illicit drug use (excluding marijuana), and homelessness.

## Discussion

Six in 10 transgender women experienced certain forms of violence and harassment during the past 12 months, and approximately one fifth reported suicidal ideation during the past 12 months. Most transgender women reported high social support from friends or significant others. Experiencing certain forms of violence and harassment and having low-moderate social support were associated with increased prevalence of suicidal ideation. Even in the presence of high family social support, certain forms of violence and harassment were still associated with higher prevalence of suicidal ideation.

The prevalence of suicidal ideation during the past 12 months in this analysis was lower than other studies among nonrandom samples of transgender persons (*2**,**16*) and young transgender women (*5*) but was disproportionately higher than a randomly selected sample of the general population in the United States (*1*). Contrary to other studies (*2**,**4**,**13**,**16**,**24**,**26*), suicidal ideation was not associated with gender-affirming therapy or poverty. Suicidal ideation was highest among those currently experiencing homelessness, consistent with other studies (*16**,**27*). The lower prevalence of suicidal ideation among those with history of incarceration >12 months ago was consistent with another study (*28*).

The prevalence of certain forms of violence and harassment during the past 12 months in this analysis was similarly high as the estimates among transgender persons in a large cross-sectional study in the United States (*2*) and in a systematic review (*8*). The prevalence of physical intimate partner abuse or harassment and sexual violence during the past 12 months in this analysis were higher than that among cisgender women in the United States (*29*), but comparable to the intimate partner violence prevalence among cisgender women of low socioeconomic status (*30*).

The analysis contributes to existing research linking certain forms of violence and harassment with increased suicidal ideation among transgender women (*13**,**16**,**17*), and these studies likewise used the gender minority stress model (*14*) to explain the findings. This model suggests that certain forms of violence and harassment are often enacted upon those nonconforming to heterosexual and cisgender norms and are underpinned by sociocultural, political, and legal marginalization of gender minorities (*2**,**8*), emphasizing the role of social determinants influencing health disparities among transgender women (*8*).

The findings in this report indicate that lack of high social support was associated with suicidal ideation, a finding consistent with other studies (*15**,**18**,**26*). However, the association between certain forms of violence and harassment with higher suicidal ideation was not moderated by social support from friends and significant others, and the association remained despite having high social support from family. Collectively, these results suggest that the association of certain forms of violence and harassment with higher suicidal ideation remained regardless of social support from any source. Mediating factors between experiences of violence and harassment and suicidal ideation (e.g., incarceration, homelessness, and poor access to education and health care) might exist such that social support alone could not adequately reduce the risk for suicidal ideation (*18*). Previous moderation studies have demonstrated mixed results (*12**,**15**,**18**,**20**,**26*). Certain studies found that the association was not moderated by social support from family (*12**,**18**,**20*) and from friends (*15**,**20*). Other studies found that social support from significant others (*15*) and parental support specific to gender identity (*26*) buffered the increased suicidal ideation associated with violence and harassment. This report contributes to limited studies exploring the relation between these variables (*12**,**15**,**18**,**20**,**26*), but this report analyzed the nuanced variables altogether (*12**,**15**,**18**,**20**,**26*).

Social support dynamics of transgender women are multifaceted. Family could be a source of social support (*31*), abuse and harassment (*32*), or both. Amid the frequent reports of rejection from family, social support from friends and significant others might fill such gaps (*33*). Moreover, the findings suggest that the effectiveness of social support as a buffer might depend on the quality and the context in which the support was provided. Not all social support might be productively helpful (*34*), and certain transgender persons report adverse experiences while receiving social support, such as microaggressions (*33**,**35**,**36*) and corumination (*18**,**37*). Microaggressions are subtle behaviors of gender-based discrimination from various perpetrators (*33*); these might even come from supportive family, friends, significant others, and persons who belong to sexual and gender minority groups (*33**,**35**,**36*). Corumination is the unproductive processing and repeated experiencing of trauma with a person who shares lived experiences (*37*). Although both were associated with poor mental health outcomes (*33**,**36**,**38*), microaggressions and corumination do not discount the protective effects of social support in general on mental health (*15**,**16**,**23*). Nonetheless, further understanding of the social support dynamics among transgender persons, including improving how researchers operationalize and measure social support, is warranted (*19*).

Addressing violence, harassment, and suicidal ideation among transgender women requires integrated multisectoral interventions (https://www.cdc.gov/suicide/pdf/preventionresource.pdf). Violence and harassment prevention can be delivered through community-led awareness and cultural changes in existing programs (*39*), such as transinclusivity in schools (*39*), homeless shelters (*40*), the criminal justice system (*8*), and health care (*8*). Holistic approaches addressing underlying socioecological factors (e.g., gender norms, economic dependence, and public attitude toward violence and harassment) have been recommended (*41**,**42*). Moreover, because transgender persons experiencing violence and harassment were more likely to access support from family, friends, and significant others than from health care providers (*43*), interventions improving the quality of social support, such as family-based interventions (*44*), life-course appropriate tools (*31*), peer-delivered support groups (*19*), and bystander engagement (*43*), could be considered. Designing interventions with the transgender community is essential because transgender persons have values and strategies (*45*) on effectively building their social capital.

## Limitations

General limitations for the NHBS-Trans are available in the overview and methodology report of this supplement (*21*). The findings in this report are subject to at least four additional limitations. First, the cross-sectional design precludes inferences on causality among violence and harassment, suicidal ideation, and social support. Second, measurement of variables might be limited by information bias. Measured violence and harassment excluded physical and verbal abuse or harassment that were not specific to their gender identity or presentation and other forms of violence (e.g., psychological and economic violence). Measured social support pertained to individual support to the participants and was not specific to structural or community levels of support. Family might pertain to family of origin or chosen family, or both; social support from significant others might be subject to nonspecificity and transientness of significant others. The survey did not assess whether sources of social support also were perpetrators of violence. Third, most data were self-reported and might be subject to recall and social desirability biases and influenced by trauma, which could underestimate the reports of suicidal thoughts and experiences of violence and harassment. Finally, the sample is not representative of transgender women residing outside of the seven urban areas. Because transgender women are hard to reach, the data might not be representative of all transgender women residing in the seven urban areas. The surveillance included an incentivized peer recruitment; therefore, participants might have been more likely to have similar characteristics, including socioeconomic status and experiences of violence (*22*).

## Conclusion

Many transgender women experience certain forms of violence and harassment and these experiences are associated with suicidal ideation. Although social support might be protective against suicidal ideation, such support does not seem to completely buffer the association between certain forms violence and harassment and suicidal ideation. Integrated and holistic approaches to violence, harassment, and suicide prevention designed by and for transgender women are needed.

## Footnotes

* 45 C.F.R. part 46.102(l)(2), 21 C.F.R. part 56; 42 U.S.C. Sect. 241(d); 5 U.S.C. Sect. 552a; 44 U.S.C. Sect. 3501 et seq.
